# Integrated mRNA and miRNA Expression Profile Analysis of Female and Male Gonads in *Acrossocheilus fasciatus*

**DOI:** 10.3390/biology11091296

**Published:** 2022-08-31

**Authors:** Wenbo Wei, Jiamei He, Muhammad Amjad Yaqoob, Lang Gui, Jianfeng Ren, Jiale Li, Mingyou Li

**Affiliations:** 1 Key Laboratory of Integrated Rice-Fish Farming, Ministry of Agriculture and Rural Affairs, Shanghai Ocean University, Shanghai 201306, China; 2 Key Laboratory of Freshwater Aquatic Genetic Resources Ministry of Agriculture and Rural Affairs, Shanghai Ocean University, Shanghai 201306, China

**Keywords:** *Acrossocheilus fasciatus*, high-throughput sequencing, transcriptome, gonad, microRNA

## Abstract

**Simple Summary:**

Gonadal development and sex differentiation are important research contents of aquaculture, which are regulated by complex and precise networks, such as mRNA–miRNA regulatory networks. *Acrossocheilus fasciatus* is an important economic fish in the south of China, and females grow faster than males. However, it is damaged by overfishing and water environment, and artificial breeding has become a concern. mRNA sequencing and miRNA sequencing were used to study the internal mechanism of sex control in *A. fasciatus*. Differentially expressed genes and miRNAs were identified and their potential biological functions were analyzed. In addition, through target gene prediction and dual-luciferase reporter assay, the interaction between 3 miRNAs and their target genes was confirmed. Our findings will contribute to the sex control of *A. fasciatus* and provide new ideas for aquaculture.

**Abstract:**

MicroRNAs (miRNAs) are regarded as key regulators in gonadal development and sex determination in diverse organisms. However, the functions of miRNAs in gonads of *Acrossocheilus fasciatus*, an economically important freshwater species in the south of China, are still unclear. Here, high-throughput sequencing was performed to investigate the mRNA and miRNAs on gonads of *A. fasciatus*. In total, 49,447 unigenes were obtained, including 11,635 differentially expressed genes (DEGs), among which 4147 upregulated genes and 7488 downregulated genes in the testis compared to the ovary, while 300 (237 known, and 63 novel) miRNAs with 36 differentially expressed miRNAs (DEMs) were identified, from which 17 upregulated and 19 downregulated DEMs. GO and KEGG enrichment analysis were performed to analyze the potential biological functions of DEGs and DEMs. Using qRT-PCR, 9 sex-related genes and 9 miRNAs were selected to verify the sequencing data. By dual-luciferase reporter assay, miR-22a-5p and miR-22b-5p interaction with *piwil1*, and miR-10d-5p interaction with *piwil2* were identified. These findings could provide a reference for miRNA-regulated sex control of *A. fasciatus* and may reveal new insights into aquaculture and breeding concepts.

## 1. Introduction

MicroRNAs (miRNAs) belong to small noncoding RNAs that are approximately 21 nucleotides in length and modulate gene expression post-transcriptionally [1]. As the universal specificity factors, miRNAs involve various biological processes through complete or partial complementary manner binding to the 3′ untranslated region of target genes to mediate gene silence [2]. To achieve these functions, miRNAs along with Argonaute proteins form the RNA-induced silencing complex (RISC) to repress target genes’ expression through mRNA cleavage, degradation, and/or translational repression [3]. In species, one miRNA can target many genes to control their expression or a single gene needs multiple miRNAs to work efficiently [4]. With miRNA’s roles identified in cell proliferation and sex determination, evidence is mounting that miRNAs are more numerous, and many of these are phylogenetically conserved [5].

Gonadal development and sex determination are the basis and prerequisite for the transmission of genetic information in vertebrates to the next generation and are also key concerns in aquaculture, which are regulated by complex and tight mechanisms, including networks between miRNAs and target genes [6,7]. In the previous studies, several sex determination genes were found in animals, such as *dmrt1* [8], *gsdf* [9], and *sry* [10]. Recently, the vital roles of miRNA in gonadal development and sex determination have been elucidated in many organisms, such as humans [11], mice [12], zebrafish [13], and medaka [14]. In mice, miR-224 is essential for self-renewal of spermatogonial stem cells by targeting DMRT1 [15], and miR-29a/b1 could regulate luteinizing hormone secretion and affect mouse ovulation [16]. In medaka, miR-202 is primarily expressed in the ovary and affects female fertility through regulating oogenesis process. Furthermore, a dramatic reduction in the number of eggs can be observed after knocking out miR-202 [17]. Moreover, miRNAs also involve germline stem cells differentiation [18], spermatogenesis [19], and granulosa cells apoptosis [20].

*Acrossocheilus fasciatus* is an important economic and ornamental fish, which is widely distributed in the gravel deposits of rivers and streams in southern China [21]. In recent years, the distribution range and quantity of *A. fasciatus* have declined rapidly as a result of overfishing and the degradation of the aquatic environment, and artificial breeding has become a new option. As females grow significantly faster and are more highly valued than males [22], all-female stocks are preferable in aquaculture. Consequently, understanding the mechanisms of sex determination and differentiation is essential for *A. fasciatus*. With the continuous expansion of the scale and demand of aquaculture, it is crucial to use omics technologies to facilitate the development of aquaculture and aquatic breeding. Recently, the speed and quality of omics technologies, such as miRNA and mRNA omics have been significantly improved. Many organisms are investigated to analyze sex determination and differentiation factors by transcriptome or miRNA analysis, including *Oreochromis niloticus* [23], *Acanthopagrus latus* [24], *Hyriopsis schlegelii* [25], *Odorrana tormota* [26], and *Hyriopsis cumingii* [27]. However, in A. fasciatus, there are few studies on the interactions between miRNAs and their target genes on gonadal development and sex differentiation. Through high-throughput sequencing analysis, we can further understand the internal processes of sex differentiation in A. fasciatus and lay a groundwork for all-female breeding.

In this study, mRNA sequencing and miRNA sequencing were performed to detect gonadal-related genes and miRNAs in gonads of *A. fasciatus*. The differentially expressed genes and miRNAs between ovaries and testes were distinguished, and miRNA binding target genes were confirmed. Furthermore, the expression patterns of genes and miRNAs in kinds of tissues were revealed by qRT-PCR. The results will advantage further research of miRNAs and target genes concerned with the reproduction of *A. fasciatus* and provide a reference for aquatic breeding.

## 2. Materials and Methods

### 2.1. Fish, Samples Collection, and RNA Extraction

Healthy and sexually mature adults of *A. fasciatus* of 12–14 cm in length and two years old were purchased from Jinhua Hengyuan Agricultural Science and Technology Co., Ltd. (Jinhua, China). After being frozen on ice, gonadal tissues (testis and ovary) and other organ tissues (eye, brain, liver, intestine, and kidney) were extracted quickly, which were divided into three parts: one part for the construction of cDNA libraries, the second part for the construction of miRNA libraries, and third part for gender identification. The blood clots on the tissues were rinsed with 1× PBS and placed into 2 mL cryopreservation tubes, which were frozen in a refrigerator at −80 °C. RNA was extracted by a TRIzol reagent (Invitrogen, Carlsbad, CA, USA) and the concentration of total RNA was detected by NanoDrop 2000 Spectrophotometer (Thermo Fisher Scientific, Waltham, MA, USA). Then, the integrity of RNA was validated through agarose-gel electrophoresis. Reverse transcription of total RNA into cDNA was performed with the usage of PrimeScriptTMFirst Strand cDNA Synthesis Kit (Takara, Kusatsu, Japan). All animal experiments were conducted by Laboratory Animal Guideline and approved by the Ethics Committee of Shanghai Ocean University.

### 2.2. cDNA Library, Small RNA Preparation, and Sequencing 

Six sequencing libraries were constructed, three libraries (Afa_O1, Afa_O2, Afa_O3) from female groups and the other three (Afa_T1, Afa_T2, Afa_T3) from male groups. The cDNA libraries were conducted using the TruSeq RNA Sample Prep Kit (Illumina, San Diego, CA, USA) in accordance with the manufacturer’s recommendations. After passing the check test of Agilent 2100 Bioanalyzer (Agilent Technologies, Palo Alto, CA, USA), six cDNA libraries were sequenced on an Illumina HiSeq X Ten platform.

Small RNA libraries were arranged to utilize around 1 μg of total RNA beneath the convention of the TruSeq Small RNA Sample Preparation Kit (Illumina, San Diego, CA, USA). After a quality test, the constructed libraries were sequenced at OE Biotech. Co., Ltd. (oebiotech, Shanghai, China) utilizing Illumina HiSeqTM 500 according to the protocol.

### 2.3. mRNA sequencing and miRNA Sequencing Analysis, and Assembly

Data processing was carried out in accordance with the methods as depicted previously with a minor modification [28]. In brief, clean reads were gotten through getting rid of low-quality reads, reads containing adapters, and reads containing poly-N from raw reads. Then, clean reads were assembled through Trinity software (http://trinityrnaseq.sf.net) accessed on 6 December 2020 according to the methods reported by [29].

For miRNA-seq, clean reads were gotten through getting rid of low-quality sequences, adapter sequences, sequences containing poly-N, and sequences less than 15 nt and greater than 41 nt. The size of small RNA is usually 18–35 nt, among which the size of miRNA is usually 21–25 nt, piRNA is usually 30 nt, while tiRNA and tRFs are usually 30–35 nt. Clean reads and alternative species sequences were compared by Bowtie or BLAST software to obtain annotated results and unannotated novel miRNAs. Unannotated miRNAs were analyzed by miRDeep2 [30]. In addition, the clean reads were aligned with the Rfam library, cDNA sequence, species repeat sequence library, and miRBase library, respectively, to obtain small RNA classification and annotation.

### 2.4. Differential Expression Analysis of mRNAs and miRNAs, Prediction and Annotation of DEGs and DEMs

mRNAs expression levels were carried out by counting FPKM (fragments per kilobase per million mapped reads) using StringTie, while miRNAs expression was counted by TPM (transcript per million). Next, the reads of mRNAs and miRNAs were standardized for superior comparison to assess the differentially expressed mRNAs and miRNAs between the male and female. The DE mRNAs and DE miRNAs were selected with expression quantity (fold change > 2 or fold change < −2) and significance of expression difference (*p* < 0.05) and visualized with the ggplot2 package of R. After obtaining differentially expressed genes and miRNAs, gene ontology (GO) annotation and Kyoto Encyclopedia of Genes and Genomes (KEGG) pathway analysis were performed to determine the biological roles or pathways. The significance of differential gene enrichment in each GO term and each pathway was calculated by hypergeometric distribution test. To understand the functions of DEMs, target prediction was performed through integrating TargetScan and miRanda [31] with parameters as follows: S ≥ 150, ΔG ≤ −30 kcal/mol and demand strict 5′seed pairing.

### 2.5. Validation of Sex-Biased mRNAs and miRNAs

Nine sex-related genes were chosen for qRT-PCR validation according to previously described methods [32] and nine miRNAs were chosen randomly for qRT-PCR to affirm the sequence data, in which *β*-*actin* and *U6* were used as reference genes, respectively. Total RNA and miRNA from seven tissues (eye, brain, liver, intestine, kidney, testis, and ovary) were extracted. The primers for qRT-PCR were planned using Primer Premier 6.0 and listed in Tables S1 and S2. ABI7500 real-time fluorescence quantitative polymerase chain reaction system and TB Green^®^PreMix Ex Taq™II (Takara, Kusatsu, Japan) were employed for qRT-PCR. All tests were assessed in triplicate, and genes’ and miRNAs’ relative expression levels were calculated with the 2^−ΔΔCT^ strategy, as described previously [33]. The difference was calculated by SPSS version 22.0. Statistically significant differences were inspected using paired *t*-test. A value of *p* < 0.05 was regarded to be measurably significant.

### 2.6. Cell Transfection and Dual-Luciferase Reporter Assay

Bioinformatics analysis indicated that miR-22a-5p and miR-22b-5p were anticipated to have two binding sites for *piwil1* 3′UTR, while miR-10d-5p was anticipated to have one binding site for *piwil2* coding sequence (CDS). To confirm the target genes of the miRNAs, corresponding reporter genes for luciferase detection were constructed. The 3′UTR containing the miR-22a-5p and miR-22b-5p binding sites of *piwil1* was amplified by PCR and inserted into the pmirGLO vector. Moreover, *piwil1* 3′UTR mutant-type (MT) reporters were built utilizing PCR with the primers listed in Table S1. Similarly, a mutant vector for miR-10d-5p target gene detection was also constructed using Hieff Mut™ Site-Directed Mutagenesis Kit (Yeasen, Shanghai, China). Sanger sequencing confirmed the accuracy of plasmids.

Human embryonic kidney 293 (HEK293) cell was cultured and transfected as portrayed already [34]. Wild-type or mutant-type plasmids and the three mimics (GenePharma, Shanghai, China) were co-transfected into HEK293 cells by utilizing FuGENE@HD (Promega, Madison, WI, USA), respectively. Dual-Glo^®^ Luciferase Assay System (Promega, Madison, WI, USA) was carried out to distinguish luciferase levels after 36 hours of co-transfection. The test was assessed in triplicate.

## 3. Results

### 3.1. Characterization of Gonadal mRNAs and miRNAs

**Figure 1:**
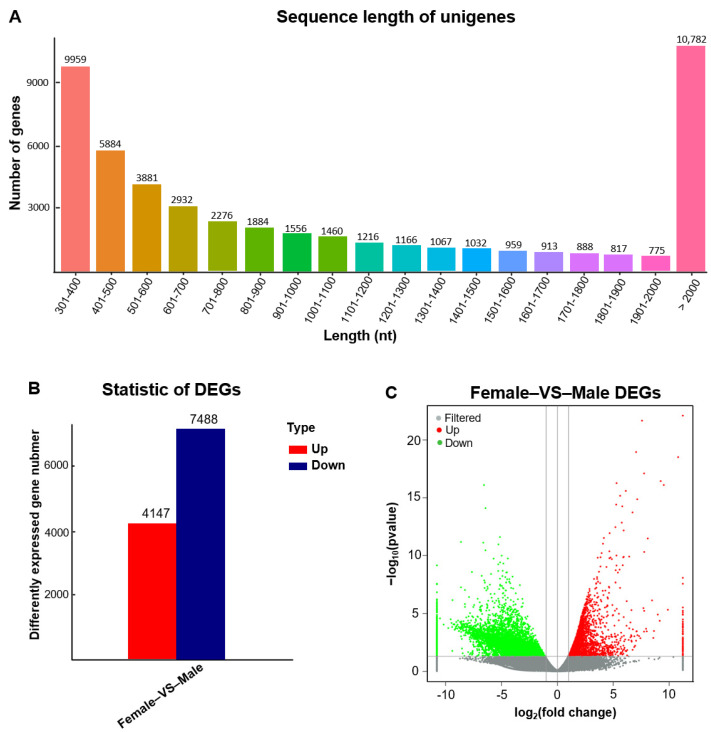
Identification of mRNAs. (**A**) Length distribution of mRNA sequences from six libraries of the *A. fasciatus*. (**B**) Statistic of differently expressed genes (DEGs) in gonads of *A. fasciatus*. (**C**) Volcanic map of female and male *A. fasciatus* differences in gonads.

The samples for mRNA-seq were sequenced from the gonads of six *A. fasciatus*, including three ovaries and three testes. A total of 291,306,108 clean reads were obtained after Cutadapt filtering with parameters as follows: removing sequences less than 15 nt or greater than 41 nt in length and other low-quality reads. The average rate of Q30 and the G + C percentage of samples were 95.67% and 47.85%, respectively (Table S3). After de novo assembly, a total of 49,447 unigenes were identified, which ranged from 301 to 16,614 with the average length is 1361.59 bp (Figure 1A). Furthermore, the annotation results of the unigenes in seven databases were NR 32,321 (65.36%), Swissprot 26,396 (53.38%), KEGG 15,493 (31.33%), KOG 19,561 (39.56%), eggNOG 28,937 (58.52%), GO 23,994 (48.52%), and Pfam 43 (0.09%) (Table S4).

**Figure 2:**
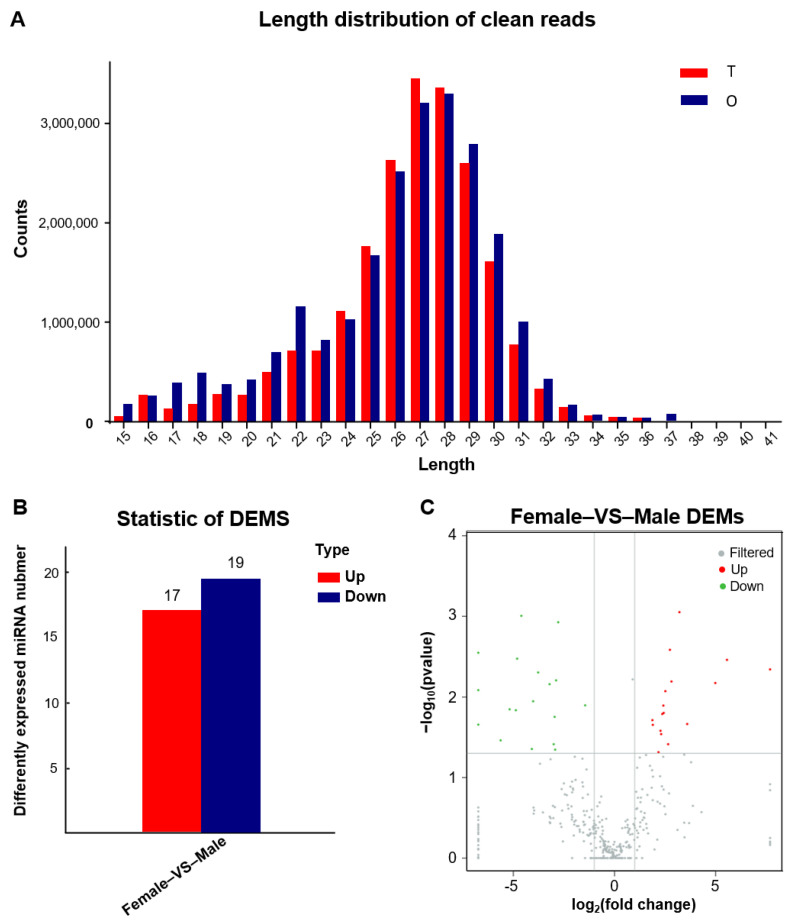
Identification of miRNAs. (**A**) Length distribution of clean reads from six libraries of the *A. fasciatus*. (**B**) Statistic of differently expressed miRNAs (DEMs) in gonads of *A. fasciatus*. (**C**) Volcanic map of differential miRNAs in female and male *A. fasciatus* gonads.

Six cDNA libraries of small RNAs from three female individuals and three male individuals were constructed. Clean reads peaked at 28 nt in ovaries, followed by 27 and 29 nt, whereas, unlike the ovaries, the peak in testes was 27 nt, followed by 26 and 28 nt. When investigating miRNAs length, the larger part of miRNAs was within the extent of 20 to 23 nt according to known miRNAs length distribution (Figure 2A). Further data analysis showed that the number of miRNAs in six libraries was 300, counting 237 known miRNAs and 63 novel miRNAs.

### 3.2. Screening for Differentially Expressed Genes (DEGs) and Functional Annotation

In total, 49,447 unigenes were obtained from A. fasciatus gonads, in which 11,635 differentially expressed genes (DEGs) were recognized between males and females, including 4147 upregulated genes and 7488 downregulated genes in male gonads compared to female gonads (Figure 1B,C). According to GO analysis, DEGs were divided into three main categories, including biological processes, cellular components, and molecular functions. In total, 10,750 DEGs showed associated GOs, and the GOs of cellular process, cell, cell part, and binding were associated with the highest number of DEGs (Figure S1). KEGG enrichment analysis indicated that DEGs were enriched to 269 pathways among which were involved in Environmental Information Processing-Signal transduction (706), Organismal Systems-Endocrine system (330), Organismal Systems-Immune system (304), and Cellular Processes-Cellular community-eukaryotes (255) (Figure S2).

### 3.3. Screening for Differentially Expressed miRNAs (DEMs) and Functional Annotation of Their Target Genes

**Figure 3:**
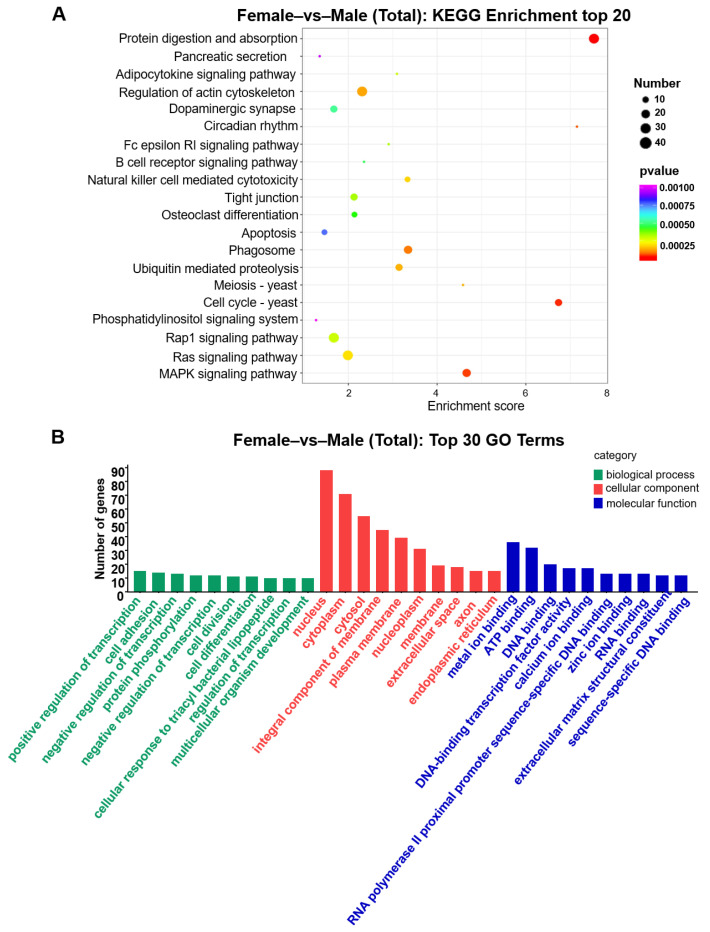
GO and KEGG analysis of target genes. (**A**) Bubble diagram of KEGG enrichment analysis. The smaller the *p*-value, the more distinctive the red color. The bigger the point is, the more genes are enriched in the pathway. (**B**) GO enrichment analysis. Top 30 GO terms of genes targeted by DEMs from *A. fasciatus* gonads. The result was divided into three categories: biological process, molecular function, and cellular component.

The miRNA libraries of *A. fasciatus* gonads were prepared and sequenced. In total, 237 known miRNAs and 63 novel miRNAs were identified. Comparing with miRNA libraries of female and male groups, 36 DEMs were obtained, including 17 upregulated DEMs (>two-fold change) and 19 downregulated DEMs in the testes compared to ovaries (Figure 2B,C); dre-let-7f, dre-let-7g, dre-let-7j, and dre-miR-122 were more highly expressed in ovaries compared with the testes, while dre-miR-124-3p was significantly downregulated (Table S5). Then, GO and KEGG annotations were carried out to explore the potential biological functions of their target genes of DEMs. GO enrichment showed that positive regulation of transcription, nucleus, and metal ion binding were the major GO terms in three categories, respectively (Figure 3B). KEGG analysis showed that more target genes were enriched in protein digestion and absorption, regulation of actin cytoskeleton, MAPK signaling pathway, and phagosome (Figure 3A).

### 3.4. Validation of miRNAs and mRNA by qRT-PCR

**Figure 4:**
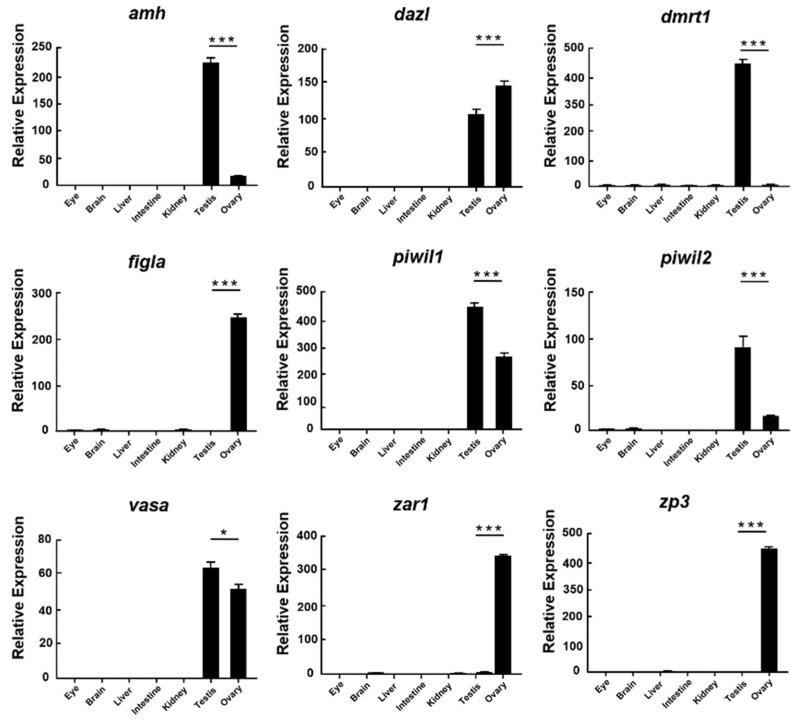
The relative expression of sex-related genes in different tissues of *A. fasciatus*. The expression was calculated through comparative CT (ΔΔCT) methods utilizing *β*-actin as a reference. *: 0.01 < *p* < 0.05; ***: *p* < 0.001.

To confirm the expression of DE genes, nine sex-related genes were chosen for qRT-PCR to verify their relative expression levels in the eye, brain, liver, intestine, kidney, testis, and ovary. The results showed that *amh*, *dmrt1*, *piwil1*, *piwil2*, and *vasa* had more tremendous expression levels in testes, while *dazl*, *figla*, *zar1*, and *zp3* had more tremendous expression levels in ovaries (Figure 4). The qRT-PCR results of these mRNAs agreed with sequence data.

**Figure 5:**
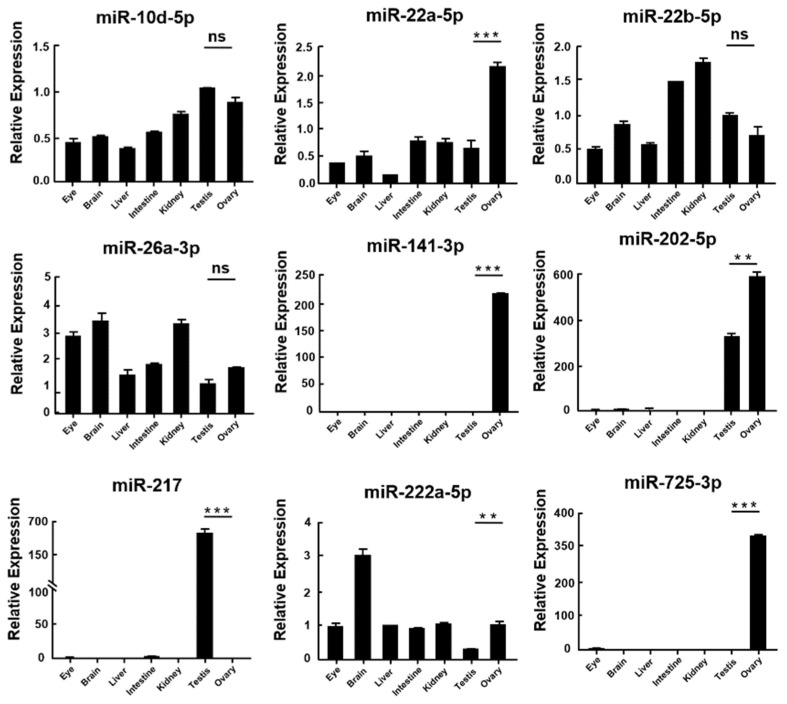
The relative expression of miRNAs in different tissues of *A. fasciatus*. The expression was calculated through comparative CT (ΔΔCT) methods utilizing *U6* as a reference. **: 0.001 < *p* < 0.01; ***: *p* < 0.001, ns: no significance.

Then, nine miRNAs were randomly chosen to investigate their relative expression levels in different tissues through qRT-PCR. The results revealed that miR-217 presented testis-biased patterns, while miR-22a-5p, miR-141-3p, miR-202-5p, miR-222a-5p, and miR-725-3p presented ovary-biased patterns. However, miR-10d-5p, miR-22b-5p, and miR-26a-3p were widely distributed in all tissues and there were no significant differences in testes and ovaries (Figure 5). The results were similar to sequencing data, showing the unwavering quality of miRNA-sequencing data.

**Figure 6:**
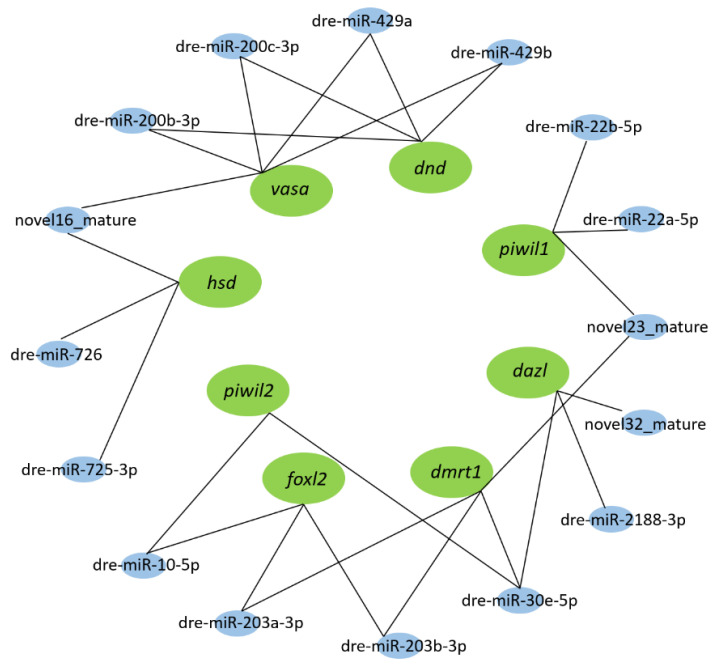
miRNA–mRNA interaction network constructed with sex-related miRNAs and genes using Cytoscape. Green represented target genes, and blue represented miRNAs. One miRNA can interact with many miRNAs. Conversely, one miRNA can bind to multiple genes.

In addition, using the bioinformatics prediction tool TargetScan and miRanda, the miRNA–mRNA interaction relationships were formulated with sex-related miRNAs and genes to explore miRNA targets in their respective unigenes. It indicated that *vasa* and *dnd* shared many miRNAs, such as dre-miR-429a, dre-miR-429b, dre-miR-200b-3p, and dre-miR-200c-3p. A similar phenomenon could be observed between *dmrt1* and *foxl2* (Figure 6). Obviously, the target genes of a miRNA or miRNAs that can be bound by a gene are diverse.

### 3.5. Piwil1 Is Regulated by miR-22a-5p and miR-22b-5p

Interaction between mRNAs and miRNAs is recognized as the basis for normal biological processes. In order to study the regulation mechanism between mRNA and miRNA during gonadal development, target gene sequences were imported into the miRNA database, using miRanda and Targetscan to predict the potential miRNAs. Three miRNAs that may be involved in gonadal development were screened. MiR-22a-5p and miR-22b-5p targeted regulation of *piwil1* by two binding sites of 3′UTR, and miR-10d-5p targeted regulation of *piwil2*.

**Figure 7:**
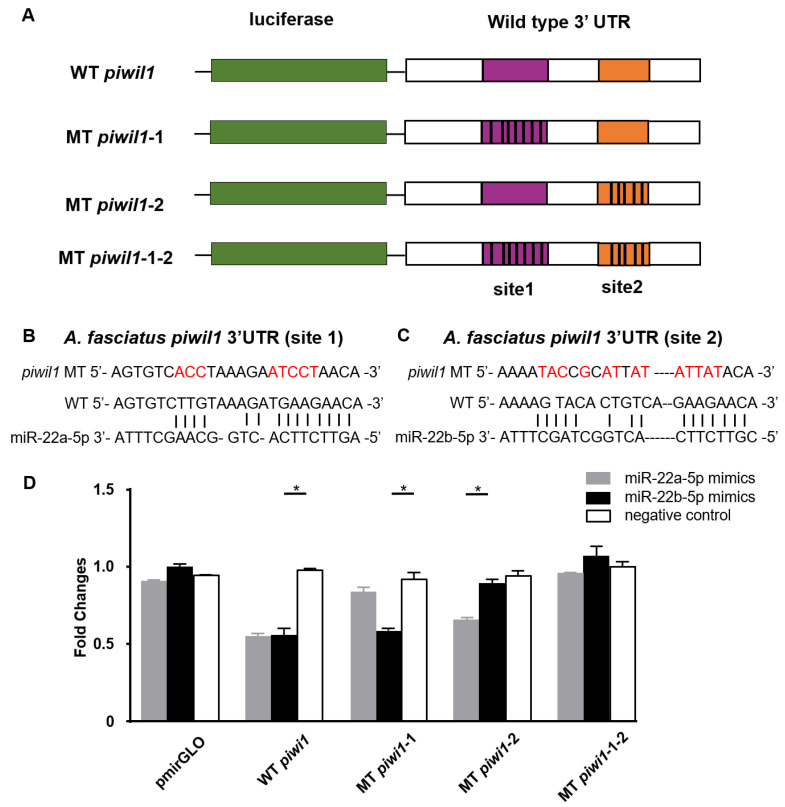
Prediction map of miR-22a-5p, miR-22b-5p, and *piwil1* target sites and the luciferase activity. (**A**) *Piwil1* target prediction map. miR-22a-5p and miR-22b-5p only have two base differences, which are basically consistent with the binding location and region of target genes. (**B**) The specific prediction diagram of miR-22a/b-5p and target genes at binding site 1 (purple area). (**C**) The specific prediction diagram of miR-22a/b-5p and target genes at binding site 2 (orange area). (**D**) The luciferase activity of miR-22a-5p, miR-22b-5p, and *piwil1*. *: 0.01 < *p* < 0.05.

To confirm the relationship between *piwil1* and miR-22-5p in vitro, 3′UTR of *piwil1* with one or two binding site mutations was inserted into the pmirGLO vector, and co-transfected into HEK293 cells with miR-22a-5p and miR-22b-5p mimics, respectively. The results indicated that the expression level of *piwil1* was suppressed due to miR-22a-5p and miR-22b-5p binding to *piwil1* 3′UTR in wild-type group. Interestingly, when co-transfected with one miRNA mimics (miR-22a-5p mimics or miR-22b-5p mimics) and the corresponding *piwil1* single mutant plasmid, the expression level of *piwil1* was significantly decreased compared with the negative control group. In addition, when both sites of piwil1 were mutated, the expression level of *piwil1* was not inhibited, that is, there was no significant difference from the negative control group (Figure 7).

### 3.6. Piwil2 Is Regulated by miR-10d-5p

**Figure 8:**
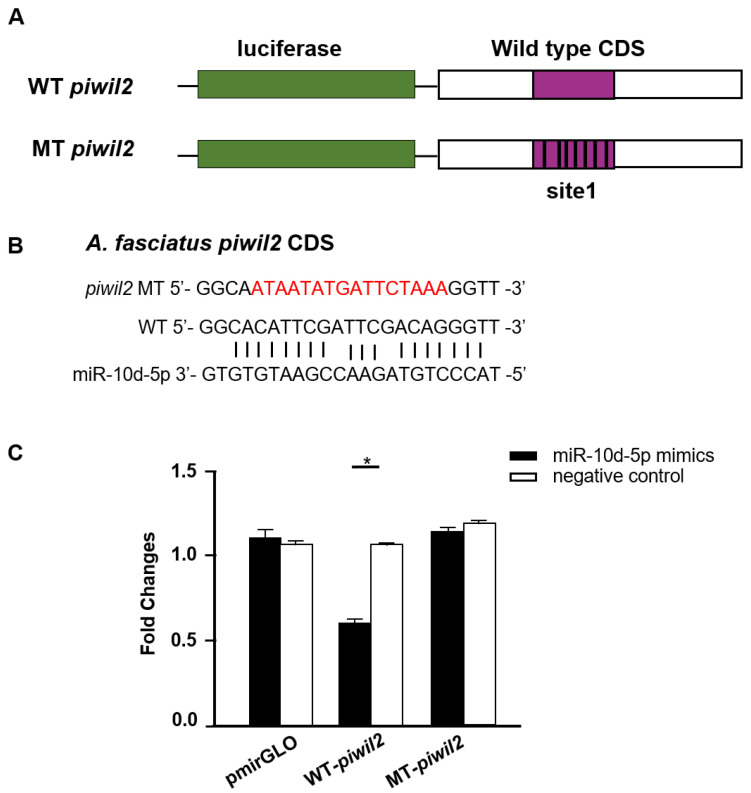
Prediction map of miR-10d-5p and *piwil2* target site and the luciferase activity of miR-10d-5p and *piwil2*. (**A**) The CDS region of *piwil2* has a targeting relationship with miR-10d-5p. (**B**) The specific prediction diagram of miR-10d-5p and target gene at the binding site. (**C**) The luciferase activity of miR-10d-5p and *piwil2*. *: 0.01 < *p* < 0.05.

*Piwil2* was predicted to target miR-10d-5p; therefore, a dual-luciferase reporter assay was also carried out to verify it. Unlike miR-22a-5p and miR-22b-5p bonded to *piwil1* 3′UTR, miR-10d-5p was predicted to bind the coding sequence (CDS) of *piwil2*. Therefore, a plasmid with *piwil2* CDS target site mutation was constructed to co-transfect with miR-10d-5p mimics into HEK293 cells. Interestingly, the relative expression level of *piwil2* was significantly decreased compared with the wild-type group (Figure 8).

## 4. Discussion

In order to investigate the mRNAs and miRNAs related to gonadal development of *A. fasciatus*, mRNA-seq and miRNA-seq analysis were carried out on testes and ovaries. In this study, mRNAs and miRNAs in gonads were identified and could provide a groundwork for screening potential miRNAs and sex-related genes, which gives a necessary knowledge into the mechanism of gonadal development in *A. fasciatus*.

Recently, much research has investigated sex-related miRNAs in female and male gonads. However, limited research has centered on the function of miRNAs in sex differentiation of commercial fish. To investigate candidate miRNAs and genes involved in gonadal development, mRNA-seq and miRNA-seq analysis were carried out. Results showed that the dominant size of miRNAs in testes and ovaries was 20–23 nt, with the typical size of Dicer-derived products, which was similar to tilapia [23], medaka [14], and *Paralichthys olivaceus* [35], while the small RNA reached a peak at 27–28 nt in both females and males according to sequence data, due to the high expression of PIWI-interacting RNAs (piRNAs) [36].

In this study, nine sex-related genes (*dazl*, *piwil1*, *piwil2*, *vasa*, *amh*, *dmrt1*, *figla*, *zar1*, and *zp3*) were identified in gonads of *A. fasciatus*, and the results of qRT-PCR showed that these genes might play important roles in gonadal development. *Dazl* persists throughout oogenesis [37] and is an integral participant in primordial germ cells formation in medaka [38]. *Piwi* is a well-studied germline-specific marker. In mice, there are three PIWI proteins, PIWIL1 (also known as MIWI), PIWIL2 (also known as MILI), and PIWIL4 (also known as MIWI2), but humans and other mammalian species have an extra PIWI gene, named PIWIL3. However, no matter which *piwi* gene it is, it plays a crucial role in diverse biological processes, such as gonadal development and gametogenesis [39]. *Vasa* could identify germ cells in gonads of medaka [33]. AMH (anti-Müllerian hormone) is associated with TGF-beta signaling pathway and further regulates the testicular differentiation in *Eriocheir sinensis* [40]. *Dmrt1* is identified as an indispensable factor of male development in medaka [41]. Furthermore, *figla*, *zar1*, and *zp3* are pivotal regulators during oocyte maturation and differentiation in diverse organisms [42,43,44]. It indicates that these genes play important roles during diverse biological activities, including gender differentiation and gonadal development. Since transcription factors are important regulators which can actuate sex determination in many animals [45], all unigenes from mRNA sequencing were aligned to the transcription factor database (AnimalTFDB), and 4430 unigenes were annotated into 69 families in the database, mainly enriched in zf-C2H2 family.

By miRNA-seq, 300 miRNAs were obtained from *A. fasciatus*, including 237 known miRNAs and 63 novel miRNAs. Bioinformatics analysis showed that 36 DEMs were identified, among which 17 miRNAs were upregulated and 19 miRNAs were downregulated in male gonads compared with female gonads. A great number of miRNAs have been studied that involve gonadal development and other biological processes. MiR-202-5p is a germ plasm-specific miRNA in zebrafish [46]. MiR-217 is first reported that might regulate the development of testis in Atlantic salmon [47]. In addition, miR-141-3p can bind to death-associated protein kinase 1 (DAPK1) and represses the apoptosis of ovarian granulosa cells in rats [48]. Based on the fact that these miRNAs play essential roles in gonadal development of different organisms, it is hypothesized that these miRNAs have similar functions in *A. fasciatus*. Therefore, qRT-PCR was performed to verify the accuracy of miRNA sequencing and identify their expression patterns in different tissues. MiR-217 had high expression levels in testes, while miR-202-5p, miR-725-3p, and miR-141-3p were plentiful in ovaries, which is similar to our sequencing data and could be further study of their roles in gonads of *A. fasciatus*.

MiRNAs participate in diverse life activities by binding to target genes to affect their post-transcriptional repression [49]. In the study, 36 DEMs were predicted to bind to 365 target genes, and several target genes were verified through qRT-PCR. Through KEGG enrichment analysis, these genes were rich in MAPK signaling pathway which is very conserved across species and involves meiosis I and meiosis II progression during female gametogenesis [50]. Remarkably, statistical family analysis of known and newly predicted miRNAs found that let-7 family and miR-10 family were the most plenteous families, which is consistent with the miR-seq in pigs [51]. While miR-10 has been mainly studied in virus infection, immunity, and cancer aspects [52,53], it needs further study in gonadal development. In addition, many sex-related genes were predicted, including *nos1*, *nos3*, *igf1r*, and *igf2r*. These results showed that a great number of sex-related miRNAs and signaling pathways not only play important roles in other well-studied species, but in *A. fasciatus*, which can be further studied. Remarkably, miR-22a-5p and miR-22b-5p could regulate *piwil1*, while miR-10d-5p regulated *piwil2* utilizing the dual-luciferase reporter assay. What is more, the two binding sites of *piwil1* 3′UTR are independent of each other, and mutation of one site will obviously cause changes in the expression of *piwil1*, indicating the diversity and complexity of mRNA-miRNA regulatory sites in organisms. Interestingly, different from most of the miRNA-mRNA binding sites in 3′UTR of target genes, miR-10d-5p regulated *piwil2* by binding to the coding sequence. Previous studies also find that several miRNAs can bind to target genes’ 5′UTR, coding sequence, and gene promoters to control gene expression [54,55] and our finding confirmed the special binding site during the miRNA–mRNA interaction.

## 5. Conclusions

In the current investigation, mRNA-seq and miRNA-seq were carried out to investigate candidate genes and miRNAs for sex determination and gonadal development in *A. fasciatus*. In total, 11,635 DEGs and 36 DEMs were obtained, which might be involved in gonadal development in *A. fasciatus* using bioinformatics analysis. Additionally, the interaction between miR-22a-5p, miR-22b-5p and *piwil1*, and miR-10d-5p and *piwil2* were identified by dual-luciferase reporter assay. The experimental research results can be used as a meaningful reference for a better understanding of the sex determination and gonadal development of *A. fasciatus* and provide new ideas for aquatic breeding.

## Data Availability

The data presented in this study are openly available in Figshare at https://doi.org/10.6084/m9.figshare.20499915, accessed on 17 August 2022.

## Supplementary Materials

The following supporting information can be downloaded at: https://www.mdpi.com/article/10.3390/biology11091296/s1, Figure S1: Comparison of upregulated genes and downregulated genes distribution at GO level; Figure S2: Distribution of DEGs at KEGG; Table S1: Gene primers were used in the study; Table S2: miRNA primers were used in the study; Table S3: Summary statistics of mRNA-seq in *A. fasciatus*; Table S4: Unigenes annotation results in different databases; Table S5: Analysis of differentially expressed miRNAs between males and females.

## Abbreviations

UTR: untranslated region; CDS, coding sequence; DEGs, differentially expressed genes; DEMs, differentially expressed miRNAs; qRT-PCR, quantitative reverse transcription-polymerase chain reaction; GO, Gene Ontology; KEGG, Kyoto Encyclopedia of Genes and Genomes; nt, nucleotide.
