# Supplementary material for: Integrated mRNA and miRNA Expression Profile Analysis of Female and Male Gonads in Acrossocheilus fasciatus
Source: Biology (Basel). 2022 Aug 31;11(9):1296. doi: 10.3390/biology11091296 (PMC9495813; doi:10.3390/biology11091296)
Supplement: Supplementary file 1 [file biology-11-01296-s001.zip › Supplementary Materials Figures.pptx]

## Slide 1
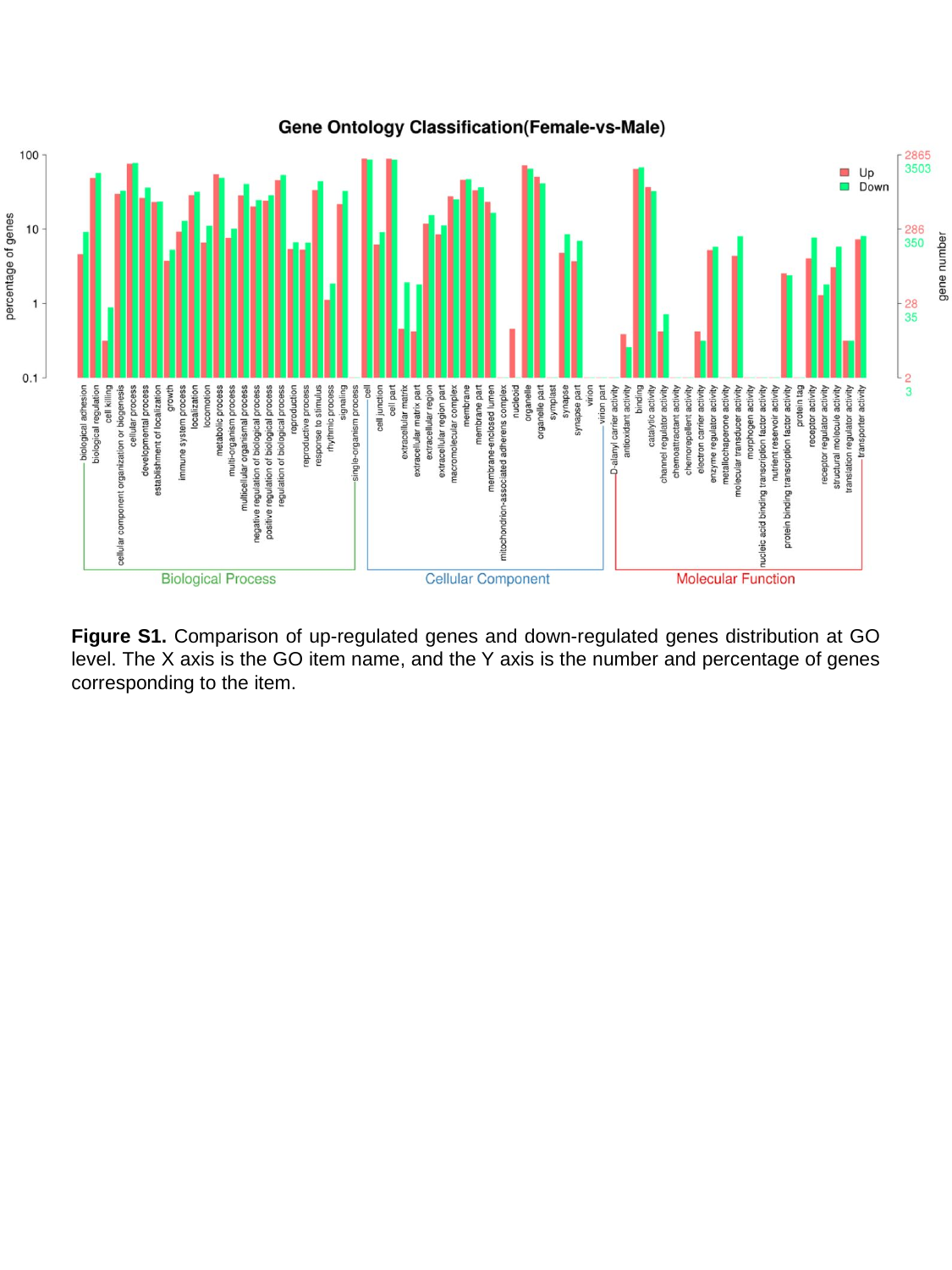

Figure S1. Comparison of up-regulated genes and down-regulated genes distribution at GO level. The X axis is the GO item name, and the Y axis is the number and percentage of genes corresponding to the item.

## Slide 2
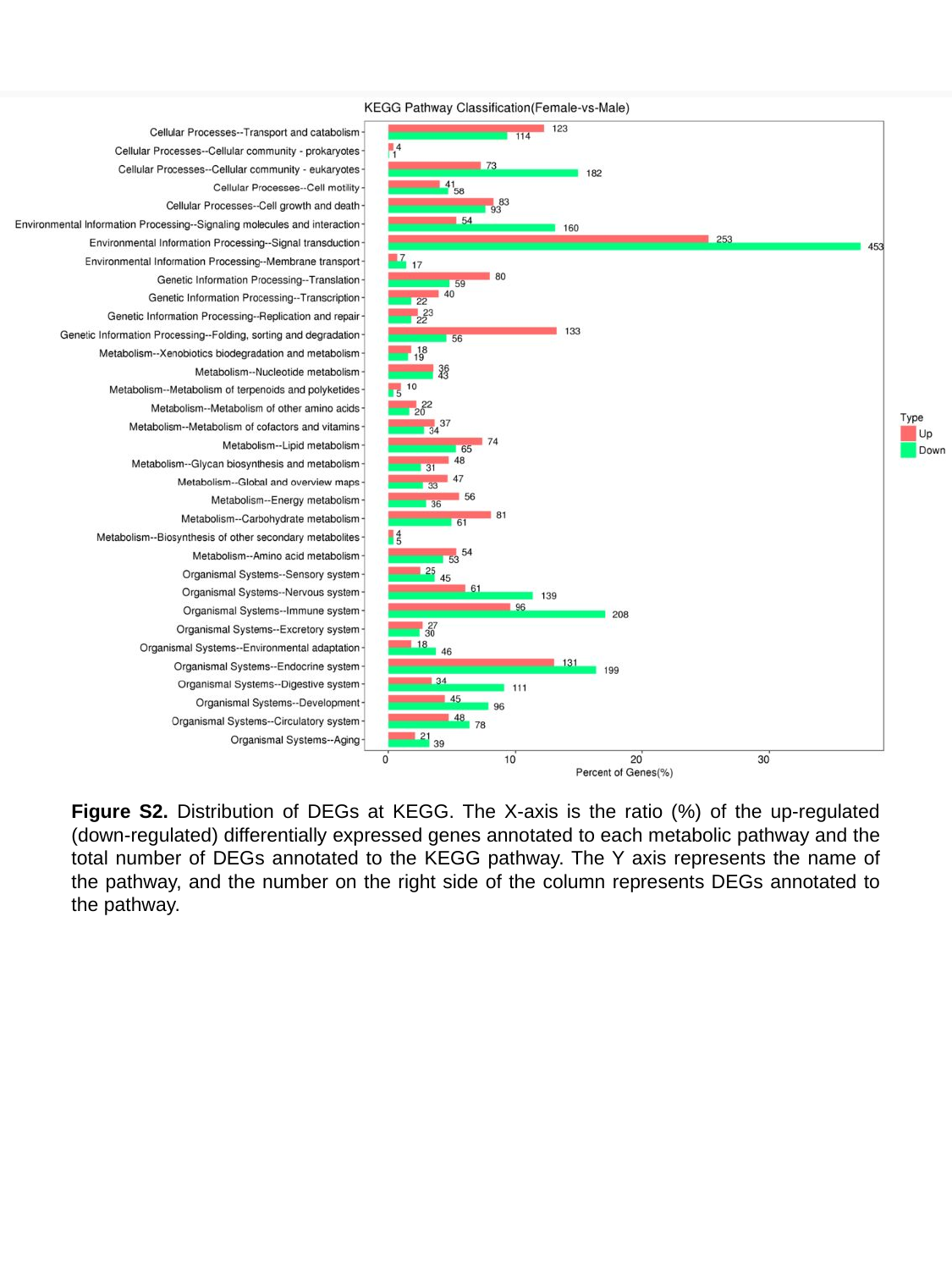

Figure S2. Distribution of DEGs at KEGG. The X-axis is the ratio (%) of the up-regulated (down-regulated) differentially expressed genes annotated to each metabolic pathway and the total number of DEGs annotated to the KEGG pathway. The Y axis represents the name of the pathway, and the number on the right side of the column represents DEGs annotated to the pathway.
