# Supplementary material for: Integrated mRNA and miRNA Expression Profile Analysis of Female and Male Gonads in Acrossocheilus fasciatus
Source: Biology (Basel). 2022 Aug 31;11(9):1296. doi: 10.3390/biology11091296 (PMC9495813; doi:10.3390/biology11091296)
Supplement: Supplementary file 1 [file biology-11-01296-s001.zip › Supplementary Materials Tables.pdf]

Table S1. Sequences primers of genes were used in the study

Primer	Sequence (5′-3′ direction)	Purpose
dazl-F	GAAGGTGGACGAGAACGAGAT	qRT-PCR
dazl-R	TGGAGATGACATGGAACGAGAA	
piwil1-F	TACCGAGATGGAGTTGGAGAC	qRT-PCR
piwil1-R	TGGACGAGTGACTTCAGTGT	
piwil2-F	GATTACCGACAGACGAGGGAGG	qRT-PCR
piwil2-R	AACCTGGGCAATCGCTGGAG	
vasa-F	CCGTGATGGTGGTAATGAAGAG	qRT-PCR
vasa-R	CTGGCTTGTCTGCTGTTGAG	
amh-F	TTCAACAGTCATTCCATCCATCC	qRT-PCR
amh-R	GACACATTGCTGCCATCTACTT	
dmrt1-F	CGCCAGCAGTGTTACCGTCTCT	qRT-PCR
dmrt1-R	AGGCATCCACCATCAGGTCAGA	
figla-F	GAATCAACTACTCCAGCAGTGAGAC	qRT-PCR
figla-R	TCTGAGCCAACCTGAACAATGTAC	
zar1-F	GCGAGGAGAGCGTGAAGGAA	qRT-PCR
zar1-R	GGACACACCACACATAAGCACTTT	
zp3-F	GTGGATGTCCTGTTGTTGCT	qRT-PCR
zp3-R	GCGTTATTGCTTACACCTTGAA	
β-actin-qF	CCTATTGAGCACGGTATTG	Internal
β-actin-qR	CTGTTGGCTTTGGGATTG	control
piwi1-3′UTR-F	TCTAGAGCATTGCAGTGACAAGTTGCAG	3′UTR
piwi1-3′UTR-R	GTCGACGCTCTTCCCAACATTTCTGATCTGT	cloning
piwil2-CDS-F	TCTAGAATGGATCCAAAGCGACCTCC	CDS
piwil2-CDS-R	CTCGAGCAGGAAGAACAGTTTCTCTG	cloning

Table S2. Sequences primers of miRNAs were used in the study

Primer	Sequence(5′-3′ direction)	Purpose
miR-202-5p-FW	GCGCGCTTCCTATGCATATACCTC	qRT-PCR
miR-202-5p-SL	GTCGTATCCAGTGCAGGGTCCGAGGTATTCGCACTGGATACGACCAAA	
miR-217-FW	GCGCTACTGCATCAGGAACTGAT	qRT-PCR
miR-217-SL	GTCGTATCCAGTGCAGGGTCCGAGGTATTCGCACTGGATACGACCCAAT	
miR-725-3p-FW	GCGCGCTTCAGTCATTGTTTCTAGT	qRT-PCR
miR-725-3p-SL	GTCGTATCCAGTGCAGGGTCCGAGGTATTCGCACTGGATACGACACTAC	
miR-141-3p-FW	GCGCTAACACTGTCTGGTAACGA	qRT-PCR
miR-141-3p-SL	GTCGTATCCAGTGCAGGGTCCGAGGTATTCGCACTGGATACGACGCATC	
miR-22a-5p-FW	GCAGTTCTTCACTGGCAAGCT	qRT-PCR
miR-22a-5p-SL	GTCGTATCCAGTGCAGGGTCCGAGGTATTCGCACTGGATACGACTAAAG	
miR-22b-5p-FW	GCCGTTCTTCACTGGCTAGCT	qRT-PCR
miR-22b-5p-SL	GTCGTATCCAGTGCAGGGTCCGAGGTATTCGCACTGGATACGACTAAAG	
miR-222a-5p-FW	GCGCTGCTCAGTAGTCAGTGTAGA	qRT-PCR
miR-222a-5p-SL	GTCGTATCCAGTGCAGGGTCCGAGGTATTCGCACTGGATACGACGGATC	
miR-26a-3p-FW	GCGCCCTATTCGGGATGACTTGG	qRT-PCR
miR-26a-3p-SL	GTCGTATCCAGTGCAGGGTCCGAGGTATTCGCACTGGATACGACGAACC	
miR-10d-5p-FW	GCGCTACCCTGTAGAACCGAATGT	qRT-PCR
miR-10d-5p-SL	GTCGTATCCAGTGCAGGGTCCGAGGTATTCGCACTGGATACGACCACAC	
U6-F	GGAACGATACAGAGAAGATTAGC	Internal
U6-R	TGGAACGCTTCACGAATTTGCG	control

Table S3. Summary statistics of the gonadal transcriptome of *A. fasciatus*

Sample	raw_reads	clean_reads	valid_bases	Q30	GC
Afa_O1	49598114	48707862	94.59%	95.46%	48.03%
Afa_O2	49000608	48129490	93.95%	95.57%	48.25%
Afa_O3	49026134	48158504	94.65%	95.58%	48.18%
Afa_T1	49712636	48777880	94.45%	95.54%	47.61 %
Afa_T2	49810260	48956150	95.51 %	95.94%	47.50%
Afa_T3	49458862	48576222	95.29%	95.98%	47.54%

Table S4. Numbers of unigenes with annotation results in different databases

Anno Database	Annotated Number	300<=length<1000	length>=1000
NR	32321 (65.36 %)	13279 (26.86 %)	19042 (38.51 %)
Swissprot	26396 (53.38 %)	8823 (17.84 %)	17573 (35.54 %)
KEGG	15493 (31.33 %)	5147 (10.41 %)	10346 (20.92 %)
KOG	19561 (39.56 %)	6119 (12.37 %)	13442 (27.18 %)
eggNOG	28937 (58.52 %)	10792 (21.83 %)	18145 (36.70 %)
GO	23994 (48.52 %)	8033 (16.25 %)	15961 (32.28 %)
Pfam	43 (0.09 %)	32 (0.06 %)	11 (0.02 %)

Table S5. Analysis of differentialy expressed miRNAs between male and female

miRNA_id	baseMean_control _Male	baseMean_case_ Female	foldChange	pval	up_down
dre-let-7f	2406.667946	22283.797475	9.259190	0.000888	Up
dre-let-7g	2588.510593	2528.188532	4.839921	0.026250	Up
dre-let-7j	2877.800496	15534.319538	5.397983	0.015753	Up
dre-miR-122	1242.193130	4619.794734	3.719063	0.022162	Up
dre-miR-124-3p	43.481124	0.408885	0.009403	0.002837	Down
